# Does Air Pollution Trigger Infant Mortality in Western Europe? A Case-Crossover Study

**DOI:** 10.1289/ehp.1002913

**Published:** 2011-01-26

**Authors:** Hans Scheers, Samuel M. Mwalili, Christel Faes, Frans Fierens, Benoit Nemery, Tim S. Nawrot

**Affiliations:** 1 Department of Public Health, Occupational and Environmental Medicine, and; 2 Department of Biostatistics, Katholieke Universiteit Leuven, Leuven, Belgium; 3 Center for Statistics, Hasselt University, Campus Diepenbeek, Diepenbeek, Belgium; 4 Belgian Interregional Environment Agency, Brussels, Belgium; 5 Centre for Environmental Sciences, Hasselt University, Campus Diepenbeek, Diepenbeek, Belgium

**Keywords:** acute effects, air pollution, case-crossover, epidemiology, infant mortality, particulate matter, SIDS

## Abstract

Background: Numerous studies show associations between fine particulate air pollutants [particulate matter with an aerodynamic diameter ≤ 10 μm (PM_10_)] and mortality in adults.

Objectives: We investigated short-term effects of elevated PM_10_ levels on infant mortality in Flanders, Belgium, and studied whether the European Union (EU) limit value protects infants from the air pollution trigger.

Methods: In a case-crossover analysis, we estimated the risk of dying from nontraumatic causes before 1 year of age in relation to outdoor PM_10_ concentrations on the day of death. We matched control days on temperature to exclude confounding by variations in daily temperature.

Results: During the study period (1998–2006), PM_10_ concentration averaged 31.9 ± 13.8 μg/m^3^. In the entire study population (*n* = 2,382), the risk of death increased by 4% [95% confidence interval (CI), 0–8%; *p* = 0.045] for a 10-μg/m^3^ increase in daily mean PM_10_. However, this association was significant only for late neonates (2–4 weeks of age; *n* = 372), in whom the risk of death increased by 11% (95% CI, 1–22%; *p* = 0.028) per 10-μg/m^3^ increase in PM_10_. In this age class, infants were 1.74 (95% CI, 1.18–2.58; *p* = 0.006) times more likely to die on days with a mean PM_10_ above the EU limit value of 50 μg/m^3^ than on days below this cutoff.

Conclusions: Even in an affluent region in Western Europe, where infant mortality is low, days with higher PM air pollution are associated with an increased risk of infant mortality. Assuming causality, the current EU limit value for PM_10_, which may be exceeded on 35 days/year, does not prevent PM_10_ from triggering mortality in late neonates.

In the past few decades, numerous studies have demonstrated that short-term exposure to elevated levels of air pollution has detrimental effects on human health. Most of these studies detected positive associations between particulate air pollution [particulate matter with an aerodynamic diameter ≤ 10 or ≤ 2.5 μm (PM_10_ or PM_2.5_)] and general mortality, or the triggering of acute cardiovascular events, especially in the elderly and people with preexisting cardiovascular and respiratory conditions (Alfaro-Moreno et al. 2007; Pope 2000; Zanobetti and Schwartz 2005).

In 1952, infant mortality doubled during the London Smog (De Angelo and Black 2008; U.K. Ministry of Health 1954), but only recently has there been renewed concern about a possible link between exposure to air pollution and children’s health. Children are considered particularly susceptible to air pollution, because their lungs and immune system are immature during the first few years of life. Prenatal exposure to elevated levels of air pollution has been associated with early fetal loss, preterm delivery, and lower birth weight (Bell et al. 2007; Schwartz 2004). Several studies have investigated the association in infants (< 1 year of age) between PM air pollution and all-cause mortality, respiratory diseases, or sudden infant death syndrome (SIDS), yielding mixed results (Hajat et al. 2007; Kaiser et al. 2004; Lin et al. 2004; Romieu et al. 2004; Tsai et al. 2006; Woodruff et al. 2008; for review, see Glinianaia et al. 2004). Most of these studies focused on urban areas in the United States or countries in transition, such as Brazil, Mexico, and Taiwan, whereas the number of studies conducted in Western Europe is very limited.

The European Union (EU) set two limit values for PM_10_ concentrations: annual mean levels of PM_10_ must not exceed 40 μg/m^3^, and daily averages must not exceed 50 μg/m^3^ on more than 35 days/year. In contrast, the World Health Organization (WHO) argues that annual averages of PM_10_ levels should not exceed 20 μg/m^3^ and that daily averages should not exceed 50 μg/m^3^ on more than 3 days/year (WHO 2006).

Using a case-crossover analysis, we investigated whether there is an association between short-term elevations of PM_10_ levels and infant mortality over a recent 9-year period (1998–2006) in the region of Flanders, Belgium, and we evaluated the effectiveness of the current EU limit values by exploring the possibility of a threshold value in the exposure–response curve. The densely populated Flemish region (> 6 million inhabitants in an area of 13,500 km², i.e., a population density of about 450 inhabitants/km²) has very low rates of infant mortality by international standards (United Nations 2007) but also among the highest concentrations of PM_10_ in Europe, as well as frequent exceedings of the prevailing EU limit values for PM_10_ (Beelen et al. 2009; Nawrot et al. 2007). Main sources of PM_10_ emission are traffic, industry, and agriculture.

In our analyses, we took into account the effect of socioeconomic status (SES), because SES has been shown to be a possible modifier of the association between air pollution and health (Carbajal-Arroyo et al. 2010).

## Materials and Methods

*Collection of data.* Mortality data. We obtained data of daily infant mortality in Flanders during the period 1998–2006 from the Flemish Agency for Care and Health (Brussels, Belgium). These data were anonymous, but the following information was provided: date of death; postal code of municipality of residence; official cause of death, according to the *International Classification of Diseases, 10th Revision* (ICD-10; WHO 1993); maturity at birth (a binary variable: mature or premature, i.e., < 37 weeks of gestation); and age at death, categorized (according to the WHO classification) as early neonatal (≤ 7 days of age), late neonatal (8–28 days of age), or postneonatal (29–365 days of age).

Air pollution data. In Belgium, PM_10_ and several other indicators of ambient air quality are continuously measured by a dense network of automatic monitoring sites (http://www.irceline.be). Nineteen of these measurement stations have been in use in the region of Flanders from 1998 on, and they are situated 25 km apart from each other on average. Using a land use regression model (Janssen et al. 2008), we calculated the daily exposure level of PM_10_ at the municipality level for each mortality case. This model provides interpolated PM_10_ values from the Belgian telemetric air quality network in 4 × 4 km grids. The interpolation is based on a detrended kriging interpolation model that uses land cover data obtained from satellite images (Corine land cover data set) (Janssen et al. 2008).

Temperature data. Temperature is a known confounder of the association between air pollution and mortality (Hajat et al. 2002; Huynen et al. 2001; Katsouyanni et al. 1997; Nawrot et al. 2007). We obtained daily average temperatures from the Belgian Royal Meteorological Institute (Uccle, Belgium). The region of Flanders is very uniform for temperature, because both altitudinal and latitudinal gradients are extremely small: Elevations range from 0 to 200 m above sea level, and the distance between the northernmost and southernmost part is only 100 km. The region is not larger than the State of Connecticut (USA). Therefore, we used temperature data from the central and representative station in Uccle (Brussels, Belgium).

Socioeconomic status. We created three classes of SES at the municipality level, based salary level, economic activity, degree of unemployment, and housing grade equipment (Dexia Bank NV 2007).

*Analytical strategy.* Case-crossover design. We investigated the association between air pollution and infant mortality using a case-crossover design, a technique developed by Maclure (1991) that combines features of the crossover design and the matched case–control design. Similar to a crossover study, each subject serves as his or her own control, and, as in matched case–control studies, the inference is based on a comparison of exposure distribution rather than the risk of disease (Jaakkola 2003). The case-crossover design is now widely used for analyzing short-term health effects of air pollution (Carracedo-Martinez et al. 2010).

**Figure 1:**
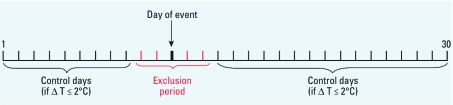
Bidirectional time-stratified case-crossover design. The timeline represents 1 calendar month (days 1–30). Only control days that were temperature matched within 2°C with the day of event were selected. ΔT, difference in temperature between day of event and control day.

Selection of hazard period and control days. We defined the hazard period, which is the brief time period when a subject is at risk, as the day of death (event day). We selected control days based on three criteria (Figure 1). First, we took control days from the same calendar month and year as the event days, both before and after the event. We chose this bidirectional time-stratified design above other selection strategies to avoid issues of bias, as explained by Janes et al. (2005) and Mittleman (2005). Second, control days and event days had to be at least 3 days apart from each other to avoid short-term autocorrelation (Levy et al. 2001). This implies a 5-day exclusion period around the event day. Third, because temperature is a known confounder of the association between air pollution and health (Hajat et al. 2002; Huynen et al. 2001; Katsouyanni et al. 1997; Nawrot et al. 2007), we selected only control days having a daily average temperature within 2°C of that on the event day. Based on this strategy, the number of control days per event ranged from zero to a maximum of 25, depending on the temperature criterion. On average, each case had 8.6 control days. Seventy-six cases (3.2%) had no control days and were, by consequence, not included in the analyses.

Shape of the association. To investigate whether there might be a threshold level in the exposure–response relationship or a plateau at higher concentrations, we studied the shape of the association between PM_10_ and risk of death by using fractional polynomials. Although linear and quadratic polynomials are commonly used, they are often inadequate to describe the shape of the association. Fractional polynomials are an alternative to classical polynomials but still fall within the realm of (generalized) linear methods. They extend the classical linear and quadratic models by allowing any power from a predefined set of values typically chosen from the set (–2, –1, –1/5, 0, 1/5, 1, 2, 3) (Royston and Altman 1994). From this family of models, the best functional form is chosen using Akaike’s information criterion (AIC). A particular feature of the fractional polynomials is that they provide a wide class of functional forms, with only a small number of terms. Moreover, the conventional linear and quadratic polynomials are included as a subset of this extended family. Based on the best-fitting model, we calculated odds ratios (ORs) for mortality in association with a 10-μg/m^3^ increase in PM_10_.

Additional analyses. To detect a possible short-term delay in the effects of exposure to PM_10_, we performed five additional case-crossover analyses with different lag structures. In these analyses, we defined the hazard period as 1, 2, or 3 days before the day of death (lag days 1, 2, and 3, respectively) or as the moving-average exposure on 2 (event day and lag day 1) or 3 (event day and lag days 1 and 2) consecutive days. We also performed a sensitivity analysis using an alternative selection strategy, with control days being matched on day of the week instead of daily temperature, thus also including the 76 cases that had no temperature-matched control day.

We conducted stratified analyses by age class, by maturity, by SES, and by cause of death, categorized as cardiorespiratory diseases (ICD-10 codes I00–J99), SIDS (ICD-10 code R95), perinatal circumstances (ICD-10 codes P00–P96), congenital and chromosomal abnormalities (ICD-10 codes Q00–Q99), or other. Infants who died from external causes (e.g., accidents, ICD-10 V00–Y98; *n* = 73) were excluded from all analyses.

Finally, we transformed the exposure value into a binary variable (i.e., below or above the EU limit value of 50 μg/m^3^) and calculated the ORs for dying on days > 50 μg/m^3^ compared with days with PM_10_ levels below that value.

Statistical analyses. Database management and statistical analyses were performed with SAS software (version 9.1; SAS Institute Inc., Cary, NC, USA). We used conditional logistic regression to evaluate the case-crossover data and to estimate the odds of all-cause and cause-specific infant mortality by exposure to PM_10_. Results are presented as ORs with 95% confidence intervals (CIs) per 10-μg/m^3^ increment in PM_10_ concentration or as the OR for days above the EU limit value of 50 μg/m^3^ against days below that value. We calculated the attributable fraction (AF) as in Steenland and Armstrong (2006). All tests were two-sided with α = 0.05.

## Results

**Figure 2:**
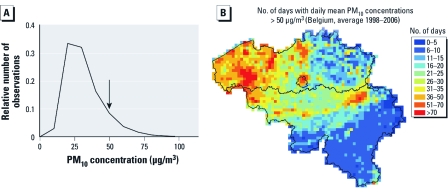
(*A*) Frequency distribution of population-weighted daily mean PM_10_ concentrations in Flanders (Belgium) during the study period (1998–2006). The arrow indicates the EU limit that may be exceeded up to 35 days/year. (*B*) Spatial distribution of population-weighted daily mean PM_10_ concentration, expressed as numbers of days with concentration > 50 μg/m^3^ (map of Belgium; the Flemish region comprises the area north of the black line, excluding the capital region of Brussels in the center of the country).

*Descriptive data.* Of the 2,455 infants who died in Flanders during the study period (1998–2006; yielding a mortality of 4.67/1,000 live births), 2,382 died from nontraumatic causes, and 1,284 infants (54%) had been born before 37 weeks of gestation. Table 1 lists ages at death and causes of death. During the study period, PM_10_ concentration averaged 31.9 ± 13.8 μg/m^3^ (Figure 2), and 321 days (an average of 35.7 days/year) had a mean daily concentration > 50 μg/m^3^ (population-weighted daily average for the whole region). For cases (*n* = 2,382), the average exposure was 32.6 μg/m^3^ (95% CI, 15.1–59.9), and on the selected control days (*n* = 20,448) PM_10_ averaged 30.7 μg/m^3^ (95% CI, 14.8–56.5). Interpolated daily average PM_10_ concentrations were strongly correlated among the 308 municipalities in the study area. Correlations ranged from 0.87 to 1.00, and the strongest correlations were among neighboring municipalities.

**Table 1:** Nontraumatic causes of death in neonates in Flanders, 1998–2006, by age class.

Table 1. Nontraumatic causes of death in neonates in Flanders, 1998–2006, by age class.								
Cause of death (ICD-10 code)		Early neonatal (≤ 7 days of age)		Late neonatal (8–28 days of age)		Postneonatal (29–365 days of age)		Total
Cardiorespiratory diseases (I00–J99)		3		3		44		50
Perinatal circumstances (P00–P96)		771		197		126		1,094
Congenital and chromosomal abnormalities (Q00–Q99)		398		140		205		743
SIDS (R95)		0		0		285		285
Others		24		22		164		210
Total		1,196		372		814		2,382

*Case-crossover analysis.* For the whole group, we found a 4% increase (95% CI, 0–8%; *p* = 0.045) in the risk of death for each 10 μg/m^3^ increase in the concentration of PM_10_ on the event day (lag day 0) (Table 2). In the sensitivity analyses with up to 3 lag days or moving-average concentrations, mortality tended to be positively associated with PM_10_ as well, but these associations were not significant (data not shown). Estimates from analyses with control days matched on day of the week were comparable to those with control days matched on temperature (data not shown). Therefore, here we report only results for exposure to PM_10_ on the event day compared with temperature-matched control days.

**Table 2:** Risk of infant death associated with a 10-μg/m^3^ increase in PM_10_ on the event day and with ambient PM_10_ concentrations > 50 μg/m^3^, stratified by age category [OR (95% CI)].

Table 2. Risk of infant death associated with a 10-μg/m^3^ increase in PM_10_ on the event day and with ambient PM_10_ concentrations > 50 μg/m^3^, stratified by age category [OR (95% CI)].								
Age category		All (*n* = 2,382)		Preterm (*n* = 1,284)		Term (*n* = 1,086)		*p*-Value for interaction*a*
OR for 10-μg/m^3^ increase in PM_10_ on event day								
All		1.04 (1.00–1.08)*		1.03 (0.98–1.08)		1.05 (0.99–1.11)		0.62
Early neonatal		1.04 (0.99–1.10)		1.03 (0.96–1.10)		1.07 (0.97–1.19)		0.49
Late neonatal		1.11 (1.01–1.22)*		1.10 (0.97–1.24)		1.13 (0.98–1.31)		0.77
Postneonatal		1.01 (0.95–1.07)		0.99 (0.88–1.10)		1.02 (0.94–1.10)		0.67
OR for days > 50 μg/m^3^ vs. days < 50 μg/m^3b^								
All		1.10 (0.94–1.29)		0.96 (0.76–1.20)		1.27 (1.01–1.61)*		0.09
Early neonatal		0.99 (0.78–1.24)		0.92 (0.69–1.22)		1.14 (0.75–1.74)		0.40
Late neonatal		1.74 (1.18–2.58)**		1.47 (0.87–2.48)		2.09 (1.15–3.79)*		0.38
Postneonatal		1.04 (0.79–1.37)		0.74 (0.43–1.27)		1.18 (0.86–1.63)		0.14
**a**Interaction between exposure and maturity at birth, with preterm birth defined as born before 37 weeks of gestation. **b**Based on EU limit value. *p ≤ 0.05; **p ≤ 0.01.								

Stratification by age class revealed stronger associations with deaths between 2 and 4 weeks of age (late neonates) than with deaths during other time periods. Specifically, a 10-μg/m^3^ increase in mean daily PM_10_ on the event day was associated with an 11% increase (95% CI, 1–22%; *p* = 0.028) in the risk of late neonatal death. In contrast, we found no evidence of effects of PM_10_ on early neonatal or postneonatal mortality. Stratified analyses revealed no significant differences in associations between PM_10_ and daily mortality among preterm versus term births (*p*-values for interaction ≥ 0.09; Table 2), although ORs were always higher for the latter group.

We further analyzed the relation between air pollution and mortality according to cause of death (Table 3). In the total group, we found no significant associations between PM_10_ and mortality from cardiorespiratory diseases or SIDS but significant associations in cases where the cause of death was perinatal circumstances. For late neonatal deaths, the associations were driven mainly by the group with congenital and chromosomal abnormalities (Table 3).

**Table 3:** Risk of infant death associated with a 10-μg/m^3^ increase in PM_10_ on the event day and with ambient PM_10_ concentrations > 50 μg/m^3^, stratified by cause of death [OR (95% CI)].

Table 3. Risk of infant death associated with a 10-μg/m^3^ increase in PM_10_ on the event day and with ambient PM_10_ concentrations > 50 μg/m^3^, stratified by cause of death [OR (95% CI)].								
Cause of death		All		Early neonatal		Late neonatal		Postneonatal
OR for 10-μg/m^3^ increase in PM_10_ on event day								
Total		1.04 (1.00–1.08)*		1.04 (0.99–1.10)		1.11 (1.01–1.22)*		1.01 (0.95–1.07)
Cardiorespiratory diseases		0.98 (0.78–1.25)		NA		NA		0.98 (0.76–1.26)
Perinatal circumstances		1.06 (1.00–1.12)*		1.06 (1.00–1.14)*		1.09 (0.95–1.25)		1.01 (0.86–1.19)
Congenital and chromosomal abnormalities		1.04 (0.97–1.12)		1.00 (0.91–1.11)		1.16 (1.00–1.35)*		1.04 (0.90–1.20)
SIDS		0.99 (0.89–1.09)		NA		NA		0.99 (0.89–1.09)
OR for days > 50 μg/m^3^ vs. days < 50 μg/m^3a^								
Total		1.10 (0.94–1.29)		0.99 (0.78–1.24)		1.74 (1.18–2.58)**		1.04 (0.79–1.37)
Cardiorespiratory diseases		0.80 (0.28–2.25)		NA		NA		0.93 (0.32–2.71)
Perinatal circumstances		1.00 (0.78–1.28)		0.96 (0.72–1.29)		1.36 (0.77–2.38)		0.77 (0.34–1.72)
Congenital and chromosomal abnormalities		1.30 (0.98–1.74)		1.03 (0.69–1.54)		2.32 (1.24–4.34)**		1.38 (0.78–2.43)
SIDS		0.94 (0.60–1.48)		NA		NA		0.88 (0.55–1.41)
NA, not applicable because of low numbers in the specified age class. **a**Based on EU limit value. *p ≤ 0.05; **p ≤ 0.01.								

Analyses that took the EU limit value of 50 μg/m^3^ as a cutoff point revealed a nonsignificant OR for the whole group but a highly significant result for late neonatal mortality, with an OR for dying on days with PM_10_ > 50 μg/m^3^ of 1.74 (95% CI, 1.18–2.58; *p* = 0.006), compared with days below the cutoff value (Table 2). The corresponding AF was 43% (15–61%). When we stratified the analysis with the EU limit value by cause of death, the highest OR (for congenital and chromosomal abnormalities) just missed significance. For other causes of death, results were also not significant. For late neonatal deaths, however, stratification by cause of death revealed a highly significant result for congenital and chromosomal abnormalities (*p* = 0.009) (Table 3).

In all settings, subanalyses of congenital malformations of the circulatory or respiratory system (ICD-10 codes Q20–Q28 and Q30–Q34, respectively) revealed similar ORs as in the whole group of congenital and chromosomal abnormalities (Q00–Q99), but because of a smaller sample size, these results did not reach statistical significance.

Analyses stratified by SES (low-, medium-, or high-SES municipality) were consistent with those estimated for the population as a whole, although ORs within SES categories were nonsignificant. ORs did not differ substantially among SES categories, as indicated by the nonsignificant interaction terms in all analyses (data not shown).

**Figure 3:**
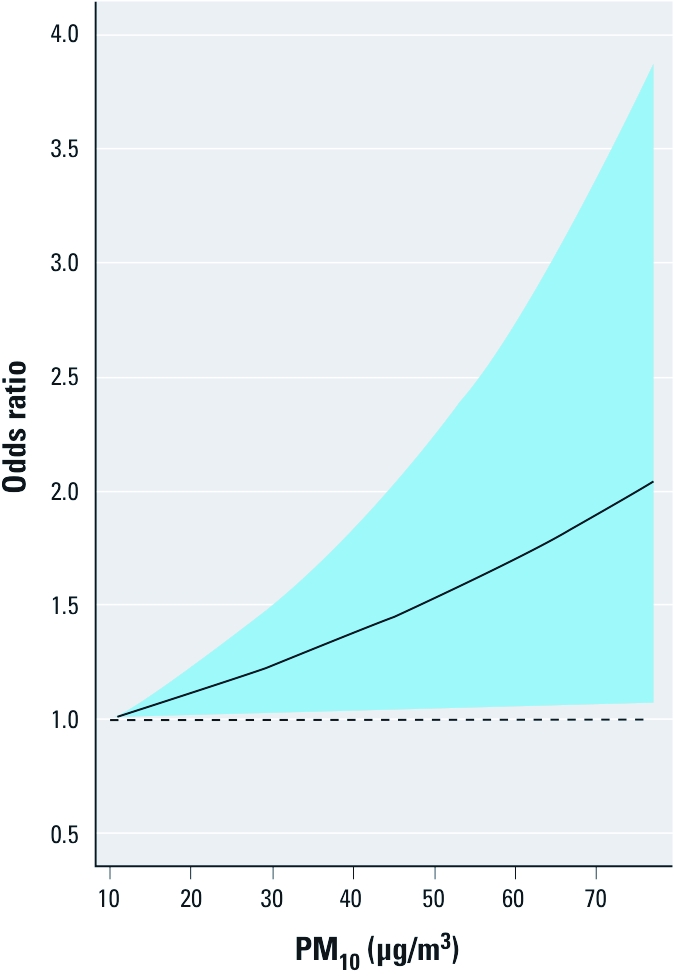
Shape of the association between exposure to PM_10_ and risk of mortality in late neonates, expressed as estimated OR with 95% CI (blue area), using fractional polynomials and 10 μg/m^3^ as reference; 77 μg/m^3^ is the 99th percentile of exposures during the study period.

In the group of late neonates, fractional polynomial analysis revealed that a linear model adequately describes the association between infant mortality and air pollution, with no evidence for a threshold or plateau (likelihood ratio test for a linear model vs. a null model; *p* = 0.030) (Figure 3). More complex fractional polynomials did not significantly improve the fit of the model, according to AIC.

## Discussion

The key finding of our study was that PM air pollution, expressed as PM_10_, is associated with late neonatal mortality, thus suggesting that airborne particles act as a rapid trigger of infant death. On days with average PM_10_ levels exceeding the EU limit value of 50 μg/m^3^—which is allowed to be exceeded on 35 days/year—the odds for late neonatal mortality was 1.74 times higher than on days below that value. Assuming causality, these results imply that on days above the EU limit value of 50 μg/m^3^, 43% (the AF) of late neonatal mortality could be triggered by an acute increase in fine PM air pollution levels on the same day. The shape of the association between the risk of late neonatal mortality and PM_10_ (Figure 3) gives no evidence for a threshold, thus suggesting the risk exists even at < 50 μg/m^3^. Analyses of lagged exposures suggested that exposure on the event day was more important than exposure during the 3 days preceding the event.

Most publications on infant mortality and PM air pollution have used a time-series approach. The case-crossover design represents a relatively novel approach to study acute health effects. It was developed in the early 1990s by Maclure (1991) to study effects of brief exposures on the change in risk of acute and discrete events, such as myocardial infarction. Recently, the case-crossover design has been applied to assess effects of short-term changes in exposure to air pollution (e.g., Romieu et al. 2004; Son et al. 2008; Tsai et al. 2006; Yang et al. 2006; Zanobetti and Schwartz 2005; for review, see Carracedo-Martinez et al. 2010). The major power of the approach is the ability to control for confounding. In the case-crossover design, all the study subjects have experienced the event. The hazard period is defined as the average time period that is relevant for the acute event, and this period is compared with control times. Thus, subjects serve as their own controls at an individual level. In contrast, the traditional time-series studies cannot control for varying individual characteristics because the unit of observation consists of daily counts of the event rather than of individuals. By matching for outdoor temperature, we excluded temperature as a potential confounder in our models, and because control days were close to event days, we controlled for seasonal effects as well (Bateson and Schwartz 1999, 2001; Maclure and Mittleman 2000). The time-stratified design for the selection of control days, as applied in our study, has been shown to be the best selection method to avoid statistical bias (Janes et al. 2005; Mittleman 2005).

So far, only five case-crossover studies on infant mortality and air pollution have been published, conducted in the cities of Seoul, South Korea (Son et al. 2008); Kaohsiung, Taiwan (Tsai et al. 2006); Taipei, Taiwan (Yang et al. 2006); Ciudad Juárez, Mexico (Romieu et al. 2004); and Mexico City, Mexico (Carbajal-Arroyo et al. 2010). Apart from the latter, they found no short-term association between postneonatal mortality and air pollution (ORs were 1.00 or 1.01 for an increase of 10 μg/m^3^). In contrast to these studies, which exclusively dealt with postneonatal mortality (> 1 month of age), we also included neonates in our analysis. We observed no evidence of an association between PM_10_ and postneonatal mortality either, but we estimated a significant positive association between a 10-μg/m^3^ increase in PM_10_ and mortality on the same day for all age classes combined that was almost entirely attributable to an association between PM_10_ and mortality during the late neonatal period (2–4 weeks after birth). In both studies performed in Mexico (Carbajal-Arroyo et al. 2010; Romieu et al. 2004), the risk of death was significantly higher in infants from low- and/or medium-SES areas than in those from high SES areas. We found no difference in ORs among municipalities classified according to SES. Because of privacy restrictions, we were not able to classify SES on an individual level, but for the present, we conclude that SES does not modify the association between PM exposure and infant death in the study region. We found no indications for a role of PM_10_ in infants who died from cardiorespiratory complications or SIDS. Earlier studies on the association between exposure to PM and SIDS yielded mixed results (Glinianaia et al. 2004; Tong and Colditz 2004), although our results for cardiorespiratory deaths may be unreliable because of the very small sample size. In the present study, we estimated the highest ORs for deaths attributed to congenital malformations and perinatal circumstances, but only the latter proved to be significant for the whole study population, and only the former for deaths among late neonates.

We did not find clear evidence of differences between term and preterm births, and we did not detect a significant association between air pollution and early neonatal mortality during the first week of life. Reasons for this might be that the most susceptible children die during the first week of life because of conditions that do not need to be triggered by air pollution, or that measured outdoor air pollution does not reflect actual exposure during the first week of life (or during the first month for premature infants), because most of these newborns probably would have remained in the hospital during this time. However, we had no access to data on the duration of hospitalization after birth to verify this hypothesis.

In this context, a limitation of our study is the use of outdoor measurements of air pollution with interpolations at the municipality level in order to estimate partly indoor personal exposures. However, recent studies (Janssen et al. 2005; Williams et al. 2000) comparing personal and ambient exposure have reported good correlations among day-to-day changes in central measurement stations of PM and personal exposure. In addition, we found very high correlations (ranging from 0.87 to 1) among municipalities for the interpolated PM_10_ levels. In other words, spatial variability in PM_10_ (which is rather low in our small study area) appeared to be less important than temporal variability, which is driven largely by weather conditions. During stable meteorologic conditions with low wind speeds, and in the presence of a temperature inversion, locally produced pollution accumulates in the lower parts of the atmosphere, which results in a cloud of dust inhaled by humans.

In their comprehensive review, Pope and Dockery (2006) discuss several plausible biological pathways for the relationship between exposure to PM and health. They derived evidence for these pathways mainly from observations on adults or experiments on animals, but at least some of the proposed mechanisms, such as pulmonary or systemic inflammation and modulated immunity, are likely to explain adverse health effects in infants as well, because their lungs, heart, and immune system are immature and fragile. In particular, there is growing evidence that ambient air pollution is associated with decreased heart rate variability (HRV) in adults (Pope and Dockery 2006), and reduction in HRV is a plausible biological mechanism in infant deaths as well (Patzak 1999). We did not find significant associations between PM_10_ and cardiorespiratory diseases as the official cause of death, but the number of children in this group was very low, which in turn might be the consequence of misclassification on death certificates. [Subtle mechanisms such as systemic inflammation or HRV are presumably more easily overlooked than perinatal or congenital abnormalities; see Nembhard et al. (2008) and references therein for examples of misclassification of cardiovascular diseases.] Hence, there is clearly a need for further research in order to understand the underlying mechanisms of the observed associations between air pollution and mortality in infants, as well as a better differentiation between acute and chronic effects of air pollution in this segment of the population.

## Conclusions

Our study shows that air pollution standards have to be taken more seriously. We estimated that 43% of mortality during the late neonatal period may be triggered by peaks of PM_10_ > 50 μg/m^3^. We do not claim that air pollution was the major, let alone the only, cause of death in these infants, but our data suggest that air pollution may precipitate death in infants with preexisting conditions. A trigger is not necessarily the primary cause of death, but it may increase the risk of death in susceptible infants, such as infants with perinatal complications or other preexisting conditions.

European regulation, which currently uses standards that are considerably higher than those of the WHO (2006), stipulates that we may have a maximum of 35 days/year with a mean PM_10_ > 50 μg/m^3^ [comparable to the U.S. Environmental Protection Agency (2011) standard for PM_2.5_ of 35 μg/m^3^ (~ 46 μg/m^3^ PM_10_)]. In Belgium, this standard is barely met at present, and although minor improvements due to emission reduction measures are expected, the limit value of 50 μg/m^3^ will continue to be exceeded frequently in the coming decade. The same is largely true for other European regions, including northern France, the southern part of the Netherlands, the German Ruhr area, and the Po valley in Italy. The argument that it is difficult to meet standards in densely populated areas ignores the fact that the importance of a factor with respect to public health increases in proportion to the number of people who are exposed to it.
